# Effects of *INSL3* and *WNT2B* gene polymorphisms on seasonal reproductive traits of Xinjiang Qira black sheep, Kazakh sheep and Duolang sheep

**DOI:** 10.1080/10495398.2025.2498677

**Published:** 2025-05-03

**Authors:** Jingdong Bi, Sulaiman Yiming, Jiaqi Li, Quanfeng Wang, Manjun Zhai, Shaoqi Cao, Mengting Zhu, Hua Yang

**Affiliations:** ^a^ College of Animal Science, Xinjiang Agricultural University, Urumqi, China; ^b^ State Key Laboratory of Sheep Genetic Improvement and Healthy Production, Xinjiang Academy of Agricultural and Reclamation Science, Shihezi, China; ^c^ Xinjiang Jinken Aoqun Agriculture and Animal, Husbandry Technology Co., Ltd., Yutian, China; ^d^ Xinjiang Uygur Autonomous Region Animal Husbandry General Station, Urumqi, China

**Keywords:** Sheep (*Ovis aries*), seasonal reproduction, litter size, *INSL3*, *WNT2B*

## Abstract

The purpose of this study was to investigate the polymorphism and genetic correlation of *INSL3* and *WNT2B* genes with seasonal estrus and litter size in three different Xinjiang sheep breeds. The genetic diversity of *INSL3* and *WNT2B* genes were analyzed, and their association with litter size and estrous traits were analyzed. The results showed that two SNPs (SNP1, SNP2) were detected in *INSL3* gene and there were three genotypes in SNP2 (*INSL3* (A100T)), named of AA, AT and TT, A was the dominant allele. Additionally, five SNPs (SNP3, SNP4, SNP5, SNP6, SNP7) were detected in the *WNT2B* gene and there were three genotypes in SNP4 (*WNT2B* (G126T)), named GG, GT and TT, G was dominant allele. SNP2 was in Hardy-Weinberg equilibrium in three sheep breeds (*P* > 0.05). SNP4 was deviated from Hardy-Weinberg equilibrium in three sheep breeds (*P* < 0.05). Further, AT genotype of SNP2 (*INSL3* (A100T)) could significantly affect the estrus trait in Duolang sheep and Qira black sheep, and related to the litter size in Duolang sheep. The *WNT2B* significantly affected the estrus and litter size of Duolang sheep and Qira black sheep. *INSL3* (A100T) and *WNT2B* (G126T) may be potential molecular markers for controlling seasonal reproductive trait in sheep.

## Introduction

Sheep (*Ovis aries*) is one of the important agricultural economic animals in China. The reproductive efficiency of sheep is a key driving force of profitability. The development of new strategies to improve reproductive efficiency has always been the focus of many researchers.^1^^,^^2^ Xinjiang was one of the second largest pastoral areas in China. Its unique geographical advantages and eating habits provide unique conditions for the development of sheep industry. In the process of long-term management of animal husbandry, Xinjiang local herdsman had the custom of nomadism and stocking. They prefer to raise these local breeds with strong adaptability, rough feeding resistance and fast growth, such as Kazakh sheep, which is a characteristic local breed in northern Xinjiang. It has fast growth and development and strong adaptability to the producing areas. However, its seasonal estrus, ovulation and lambing are the main reasons for the low efficiency of breeding industry, the reproductive rate of adult ewes was 102%.^3^ Duolang sheep was a characteristic local breed in southern Xinjiang. It has the characteristics of large body size, more meat production, perennial estrus, high reproduction rate and stable heredity, the average lambing rate was 113%−130%.^4^ Qira black sheep was also a characteristic local breed in southern Xinjiang, which was famous for its perennial estrus and high prolificy. Qira black sheep have dense lambing characteristics, with an average lambing rate was 215.46%.^5^^,^^6^ Therefore, it was important to study the relationship between polymorphism of genes related to reproductive traits and litter size and estrus traits of Xinjiang native sheep, to understand the genetic basis of these genes in the reproductive process, which played an important role in improving and breeding varieties.

Reproductive trait was usually considered to be the most important indicators among the three major economic traits of sheep. Seasonal reproduction was a reproductive phenomenon that ewes only show in a specific season to adapt to the needs of natural selection.^7^ The estrus of sheep is accompanied by changes in the length of sunshine, which belongs to the short-day estrus animals. It is mainly expressed in autumn and winter. Its seasonal estrus will enable offspring to be born in a suitable environment, so natural conditions are conducive to improving the survival rate of offspring. The estrus identification was usually carried out by ram test, blood reproductive hormone detection and B-ultrasound technique. Under the regulation of external environment and internal genes, the heritability is low to medium.^8^ According to previous studies, *INSL3* and *WNT2B* genes may be functional genes that play a key role in mammalian reproduction.^9^^,^^10^ Combined with the previous data and the relevant literature, it was found that *INSL3* and *WNT2B* genes may be related to the reproductive traits of sheep.^3^^,^^6^^,^^9^ *INSL3* may be involved in the differentiation of germ cells and affect the reproductive performance of mammals, also *WNT2B* may affect the proliferation and differentiation of germ cells through the Wnt signaling pathway.^11^^,^^12^ Researches had also been proposed, *INSL3* and *WNT2B* were the important candidate genes affecting sheep reproductive traits.^13^^,^^14^ Testosterone encoded by *INSL3* gene has an important effect on the reproductive traits of sheep, including testicular development, spermatogenesis, sex hormone levels and estrus. *INSL3* encodes a relaxin-like hormone, which is secreted by Leydig cells and specifically binds to the transmembrane receptor encoded by *RXFP2.*^15^ *INSL3* regulated the process of testicular descent during embryonic period by binding to RXFP2 receptor, which was a key step in the development of male internal and external reproductive organs. *INSL3* also regulates the function of Leydig cells in adult males and affects the synthesis and secretion of testosterone. In adult female mammals, the circulating *INSL3* concentration is much lower than that in males.^16^ Anand detected the expression of *INSL3* in sheep blood by specific enzyme-linked immunosorbent assay, and found that the expression of *INSL3* in non-pregnant ewes was 4 times that of pregnant ewes.^16^ The polymorphism of *INSL3* gene may be significantly associated with traits such as gonad maturation. Ivell’s study found that *INSL3* concentrations were significantly lower in all three hypogonadism categories compared to normal gonadal subjects.^17^ In addition, it was found in the study that *WNT2B* was the target of miR-324-3p, and the overexpression of miR-324-3p inhibited the expression of *WNT2B.*^18^ *WNT2B* gene was involved in regulating uterine development and endometrial gland formation in newborn sheep.^19^ Furthermore, *WNT2B* with c.802 C > T:p.(Arg268Cys) mutation could inhibit differentiation of the progenitor cells in the marginal retina by downregulating the expression of proneural genes, affecting mammalian reproductive function.^20^ It could be inferred that there was a certain correlation between *WNT2B* gene and sheep reproduction. Therefore, it was of great significance to study the correlation between *INSL3* and *WNT2B* genes and reproductive traits in sheep, so as to further understand the reproductive mechanism of sheep and improve production performance.

In this experiment, Xinjiang local Kazakh sheep, Qira black sheep and Duolang sheep were used as the experimental samples. The single nucleotide polymorphisms (SNP) of *INSL3* and *WNT2B* genes were detected by Sanger sequencing and PCR-SSCP technology. The genetic variation of *INSL3* and *WNT2B* genes in three sheep populations with different reproductive traits was studied, then its relationship with seasonal estrus traits and litter size traits was analyzed, which lay the foundation for finding genetic markers of reproductive traits in different breeds of sheep.

## Materials and methods

### Ethical statement

All animal treatments were according to the recommendation of the Regula-tions for the Administration of Affairs Concerning Experimental Animals of China, and approved by the Animal Care Committee of Xinjiang Agricultural University responsible for overseeing the ethical use of animals in research within the university. All methods are reported in accordance with ARRIVE guidelines for the reporting of animal experiments (2024004).

### DNA sample and reproduction traits data collection

The experimental materials were 374 sheep genomic DNA samples. Among them, 114 4-year-old female Qira black sheep with similar body weight (34.02 ± 1.28 kg) were collected from Xinjiang Jinken Aoqun Agriculture and Animal Husbandry Technology Co., Ltd. 126 4-year-old female Kazakh sheep with similar body weight (47 ± 1.36 kg) were collected from Zhaosu Saima Kazakh Sheep Breeding Farm. 134 4-year-old female Duolang sheep with similar body weight (44.12 ± 2.72 kg) of were collected from Maigaiti County Sheep Breeding Farm. Data on seasonal reproduction traits were based on breeding records from Sheep Farm. The estrous behavior was determined by the ram trial method. The data of lambing parity were obtained from the breeding archives of farm, and breeding season onset/offset identified via hormonal ELISA assays combined with estrus behavior monitoring. Finally, genomic DNA was extracted from blood sample DNA isolation kit (Tiangen, China).

### Primer design and Single-strand conformation polymorphismline (SSCP) analysis

The primers used in this study were designed with reference to the sheep *INSL3* (Gene ID: NC_056058.1), *WNT2B* (Gene ID: NC_056054.1), sequences published in GenBank. In order to amplify the sheep *INSL3* and *WNT2B* genes, two pairs of primers were designed according to the reported sheep gene sequence (*INSL3*, NC_056058.1), and five pairs of primers were designed according to the sheep gene sequence (*WNT2B*, NC_056054.1). The PCR primers were designed with Primer Premier 5.0. Table 1 shows the primer sequence information. The primers were synthesized by Sangon Biotech (Shanghai, China).

**Table 1.** Primers information.

Gene	Primers	Amplified regions (start-end)	Primer Sequence (5’–3’)	Lenght/bp	Tm/°C
*INSL3*	P1	Exon 1 (5,095,282-5,095,670)	F: TACGGTGGCTGGAAGGACAAC	286	58
			R: CGGTTTCATGGTGCTGTGTGG		
	P2	Exon 2 (5,096,488-5,097,278)	F: TACGGTGGCTGGAAGGACAAC	274	58
			R: GCTGTGTGGCATCAGGAGTTC		
*WNT2B*	P3	Exon 1 (89,923,202-89,923,591)	F: TGAAGTAGGAGCAGCCTGAGTAC	242	58
			R: CAGCAGCAGTAGCAGCAACAG		
	P4	Exon 2 (89,929,491-89,929,711)	F: TGCCAGCGTTACCCAGACATC	144	57
			R: GAGCATGACACGGCCAAAGAC		
	P5	Exon 3 (89,930,750-89,931,027)	F: TGTAGCCAGGGTGAACTGAGTG	208	60
			R: TGCGACCACAGCGATTGTTATG		
	P6	Exon 4 (89,931,686-89,931,950)	F: GCGGTTTCTGAAGCTGGAGTG	235	59
			R: GTAGTCTGGGGAGTTGTCAAAGTAG		
	P7	Exon 5 (89,937,100-89,938,330)	F: ATACCTCACTCATCCCTCCACTTC	281	57
			R: GACTCCTACCTCTTCCACTCTCC		

The PCR was performed in a 15 μL reaction volume containing 1 μL of DNA (50 ng), 1 μL of each primer, 7.5 μL of PCR Mix and 6 μL of water. The PCR system was 95 °C for 2 min, followed by 36 cycles of 94 °C for 30 s, Tm°C annealing for 30 s and 72 °C for 45 s (TA for all markers are presented in Table 1). Additionally, the authenticity of SNPs was verified by PCR-SSCP.

### Protein structure prediction of SNPs in INSL3 and WNT2B genes

The DNA sequences were translated into amino acid sequences online using novopr, and the resulting amino acid sequences were used to predict the tertiary structure of the proteins before and after the mutation of the exon region by online software (https://swissmodel.expasy.org/interactive/PU4cmH/templates/).

### Statistical analysis

Statistical analysis was performed using SAS software (version 9.2, SAS Institute Inc.), and statistical significance was defined at *P* < 0.05. A logistic regression analysis was conducted to investigate the association between the *INSL3* and *WNT2B* polymorphism with litter size and esturs trait. The potential effects of factors such as parity, lambing season, and factor interaction were evaluated. If they were found to be insignificant, they were excluded from the analysis. SPSS 20.0 (IBM Corp., Armonk, NY, USA) was used to calculate the gene frequency, genotype frequency, genetic heterozygosity and genetic homozygosity of gene SNP loci. Chi-square test was used to check whether the genotype of each mutation site conformed to Hardy-Weinberg equilibrium, and the difference of genotype of each candidate locus between different varieties was explored. The model refers to the previous results of the project team to construct different models.^21^ The association between different genotypes and litter size was analyzed using a logistic regression model: Y_ijkl_ = μ + S_i_ + P_j_ + G_k_ + F_l_ + S_i_ × G_k_ + P_j_ × G_k_ + S_i_ × P_j_ + e_ijkl_, where Y_ijkl_ was the phenotypic value of litter size; μ was the overall mean; S_i_ was the fixed effect of the i^th^ lambing season (i = 1,2,3,4); P_j_ was the j^th^ fixed effect of parity (j = 1,2,3); G_k_ was the fixed effect of the k^th^ genotype (k = 1,2,3); F_l_ was the l^th^ fixed effect of farm (l = 1,2); S_i_ × G_k_ was the interaction of season with genotype; P_j_ × G_k_ was the interaction of parity with genotype; S_i_ × P_j_ was the interaction of season with parity; and e_ijkl_ was randomized residuals. In addition, the relationship between polymorphism and seasonal estrus was analyzed using a logistic regression model: y = µ+G + S + B + e, where Y was the phenotypic value of estrus, G was a fixed effect of genotype, S was a fixed effect of season, B was a fixed effect of breed, and e was a random residual.

## Results

### PCR-SSCP and sequence analysis

**Figure 1.**
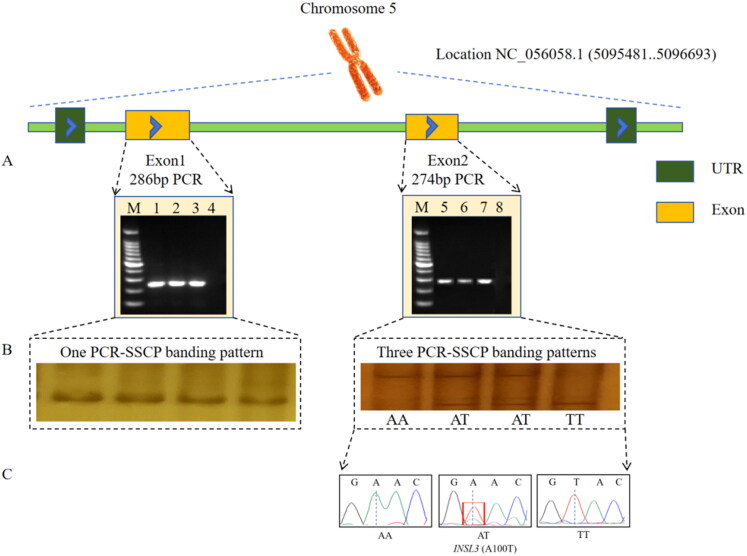
An illustration of the PCR-SSCP technique using the *INSL3* gene. (A) PCR-specific primers were designed to amplify 286 bp, and 274 bp in exon 1 and exon 2, respectively. M was DNA ladder (cat: 2029), 1,2,3 was Pl, 5,6,7 was P2,4,8 was native control. (B) PCR-SSCP results. (C) PCR product sequencing peak diagram of P2.

**Figure 2.**
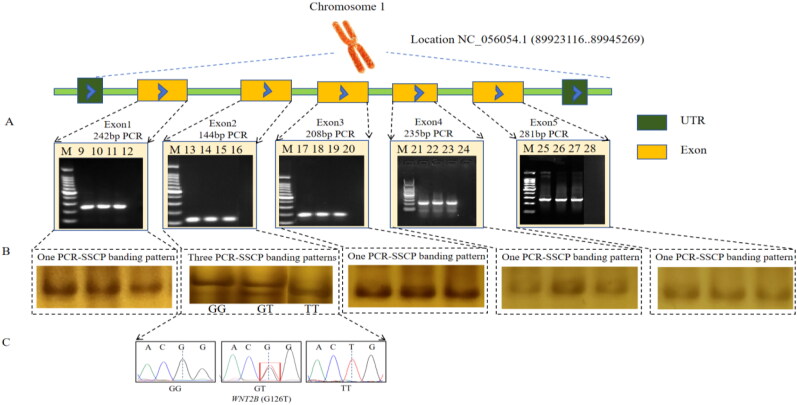
An illustration of the PCR-SSCP technique using the *WNT2B* gene. (A) PCR-specific primers were designed to amplify 144 bp, 208 bp, 281 bp, 235 bp and 242 bp in exon 1, exon 2, exon 3, exon 4 and exon 5, respectively. M was DNA ladder (cat: 2029); 9,10,11 was P3; 13,14,15 was P4; 17,18,19 was P5; 21,22,23 was P6; 25,26,27 was P7; 12,16,20,24 and 28 were native control. (B) PCR-SSCP results. (C) PCR product sequencing peak diagram of P4.

The PCR products of *INSL3* (Fig. 1A) and *WNT2B* (Fig. 2A) genes were detected by PCR. The results showed that the PCR product size was consistent with the expected results and the specificity was good. As shown in Fig. 1, Sanger sequencing of the *INSL3* gene detected a SNP2 in the exon 2 region as a synonymous mutation (Fig. 1C). Using the PCR-SSCP method, two pairs of primers for the *INSL3* gene were verified. The results showed that there were three genotypes in exon 2, named AA, AT and TT (Fig. 1B). As shown in Fig. 2, Sanger sequencing of the *WNT2B* gene detected a SNP4 in the exon 1 region as a missense mutation (Fig. 2C). The PCR-SSCP method was used to verify the two pairs of primers for the *WNT2B* gene. The results showed that there were three genotypes in exon 1, named GG, GT and TT (Fig. 2B). No SNP was detected in the remaining fragments. So subsequent association analysis was performed only for SNPs with polymorphisms.

### Genetic polymorphism analysis of INSL3 (A100T) in different sheep breeds

Genotype frequency, allele frequency, heterozygosity (H), number of effective alleles (Ne), polymorphism information content (PIC) and Shannon’s information index (S) of *INSL3* (A100T), as shown in Table 2. There were three genotypes AA, AT and TT in all three sheep breeds. The trend of genotype frequency was similar in Duolang and Qira black sheep, PIC was the same in both breeds, AT and TT were the highest and lowest frequencies, respectively. A was the dominant allele in Kazakh sheep. In the Kazakh sheep population, AA and TT were the genotypes with the highest and lowest frequencies, respectively. Although the A allele was dominant in Kazakh sheep. However, *INSL3* (A100T) was in Hardy-Weinberg equilibrium in Kazakh sheep (*P* > 0.05).

**Table 2.** Population genetic analysis of SNP2 (*INSL3* (A100T)) in different sheep breeds.

Group	Breed	Genotype frequency			Allele Frequency		MAF	H	Ne	PIC	S	Chi-squared test	P
		AA	AT	TT	A	T							
1	KAS	0.47	0.38	0.15	0.66	0.34	0.34	0.45	1.82	0.35	0.64	2.94	0.09
2	DL	0.26	0.55	0.18	0.54	0.46	0.46	0.5	1.99	0.37	0.69	1.44	0.23
3	CL	0.31	0.53	0.16	0.58	0.42	0.42	0.49	1.95	0.37	0.68	1.00	0.32

KAS, Kazakh sheep, DL, Duolang sheep, CL, Qira black sheep, H, heterozygosity, MAF, minor allele frequency, Ne, number of effective alleles, PIC, polymorphism information content, S, Shannon Information Index.

### Genetic polymorphism analysis of WNT2B (G126T) amplified loci in different sheep breeds

There were three genotypes in all breeds, named GG, GT and TT. In Kazakh sheep, Qira black sheep and Duolang sheep, GG genotype frequency was the highest, followed by GT, TT was the lowest. G was the dominant gene. The genotype frequencies of the three breed populations showed similar trends. Chi-square test showed that *WNT2B* (G126T) was deviated from Hardy-Weinberg equilibrium in all breeds (*P* < 0.05) (Table 3).

**Table 3.** Population genetic analysis of SNP4 (*WNT2B* (G126T)) in different sheep breeds.

Group	Breed	Genotype frequency			Allele Frequency		MAF	H	Ne	PIC	S	Chi-squared test	P
		GG	GT	TT	G	T							
1	KAS	0.6	0.3	0.1	0.75	0.25	0.25	0.37	1.59	0.3	0.56	4.41	0.04
2	DL	0.54	0.32	0.15	0.69	0.31	0.31	0.43	1.74	0.33	0.62	7.58	0.01
3	CL	0.57	0.31	0.11	0.73	0.27	0.27	0.39	1.65	0.32	0.58	5.49	0.02

### Tertiary structure prediction of INSL3 (A100T) and WNT2B (G126T)

**Figure 3.**
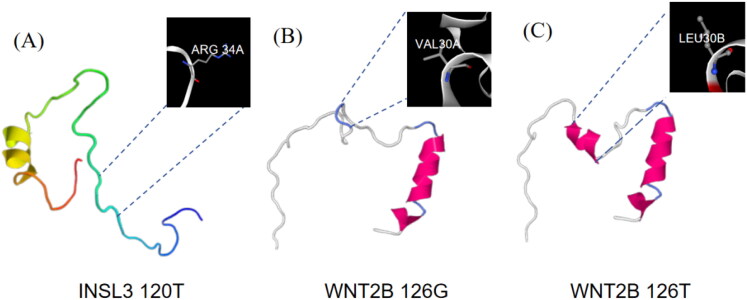
Protein structure prediction of *INSL3* and *WNT2B* genes. (A) The tertiary structure of the *INSL3* protein after mutation, which had not changed. (B) The tertiary structure of the before *WNT2B* protein before mutation. (C) The tertiary structure of the *WNT2B* protein after mutation, and its amino acid structure has changed.

The results showed that there was a synonymous mutation at *INSL3* (A100T) but its amino acid structure did not change (Fig. 3A). While the *WNT2B* gene has a G126T missense mutation, and the valine (V) (Fig. 3B) in the encoded amino acid sequence is mutated to leucine (L) (Fig. 3C). Valine and leucine are two different amino acids, which are different in chemical structure and properties. Val is a hydrophobic amino acid with a carbon chain in its side chain, while leucine is a hydrophilic amino acid with a methyl and an isopropyl group in its side chain.

### Association of INSL3 and WNT2B SNPs with litter size and seasonal reproduction

Association analysis showed that the litter size of *INSL3* (A100T) in Duolang sheep with AA and AT genotypes was significantly higher than that of TT genotype (*P* < 0.05), and the litter size of *INSL3* (A100T) in Qira black sheep with AT genotype was significantly higher than that of AA and TT genotype (*P* < 0.05). There was no significant difference in the genotype of *INSL3* (A100T) locus in Kazakh sheep population. Thus, for *INSL3* (A100T), AA and AT genotypes were favorable genotypes for Duolang and AT genotypes were favorable genotypes for Qira black sheep. The litter size with GG genotyped individuals *WNT2B* (G126T) was significantly higher than that of GT and TT genotyped individuals in Kazakh sheep (*P* < 0.05). The litter size with GT genotype was significantly higher than that of GG and TT genotypes in Duolang sheep and Qira black sheep (*P* < 0.05). In addition, The AT and AA genotypes of *INSL3* (A100T) were significantly associated with estrus in Duolang sheep, and there was no significant difference between AT and AA genotypes. However, there was no significant difference between all genotypes of *INSL3* (A100T) locus and seasonal reproduction in Kazakh sheep and Qira black sheep. The GG genotype of *WNT2B* (G126T) locus was significantly correlated with seasonal estrus in Kazakh sheep population. The GT and GG genotypes of *WNT2B* (G126T) locus were significantly correlated with seasonal estrus in Duolang sheep population. There was no significant difference between GT and GG genotypes. However, there was no significant difference between all genotypes of *WNT2B* (G126T) locus and seasonal reproduction in Qira black sheep population. In summary, there were differences in seasonal reproductive traits between seasonal estrus sheep breeds and perennial estrus sheep breeds (Table 4).

**Table 4.** Association analysis between genotypes of SNP2 and SNP4 with litter size and seasonal reproduction.

Gene locus	Breed	Genotype	Litter size	Estrus
*INSL3* (A100T)	KAS	AA	1.31 ± 0.06(P = 0.071)	1.15 ± 0.04 (P = 0.070)
		AT	1.21 ± 0.06 (P = 0.062)	1.10 ± 0.05 (P = 0.071)
		TT	1.11 ± 0.10 (P = 0.074)	1.16 ± 0.08 (P = 0.066)
	DL	AA	1.98 ± 0.08a (P = 0.033)	1.86 ± 0.04a (P = 0.031)
		AT	2.13 ± 0.06a (P = 0.026)	1.99 ± 0.03a (P = 0.028)
		TT	1.29 ± 0.12b (P = 0.060)	1.10 ± 0.05b (P = 0.061)
	CL	AA	1.93 ± 0.11b (P = 0.054)	1.87 ± 0.06 (P = 0.066)
		AT	2.52 ± 0.08a (P = 0.032)	1.94 ± 0.04 (P = 0.064)
		TT	1.90 ± 0.13b (P = 0.052)	1.76 ± 0.07 (P = 0.058)
*WNT2B* (G126T)	KAS	GG	1.42 ± 0.05a (P = 0.028)	1.25 ± 0.04a (P = 0.034)
		GT	1.10 ± 0.06b (P = 0.066)	1.04 ± 0.05b (P = 0.060)
		TT	1.00 ± 0.09b (P = 0.069)	1.00 ± 0.07b (P = 0.061)
	DL	GG	1.98 ± 0.08a (P = 0.036)	1.88 ± 0.05a (P = 0.023)
		GT	2.13 ± 0.06a (P = 0.024)	1.94 ± 0.04a (P = 0.030)
		TT	1.29 ± 0.12b (P = 0.053)	1.19 ± 0.06b (P = 0.058)
	CL	GG	2.07 ± 0.12b (P = 0.062)	1.80 ± 0.06 (P = 0.061)
		GT	2.46 ± 0.08a (P = 0.031)	1.95 ± 0.04 (P = 0.053)
		TT	1.90 ± 0.14b (P = 0.063)	1.81 ± 0.07 (P = 0.066)

Note: Different lowercase letters indicate significant differences (*P* < 0.05).

## Discussion

Litter size was an important determinant of sheep industry efficiency and productivity, and there are differences between different breeds. Different sheep breeds had different estrus patterns, which were generally divided into two types: seasonal estrus and perennial estrus. Seasonal estrus sheep, usually estrus in spring and autumn or autumn and winter, are affected by environmental factors such as light, temperature and nutrition. Sheep that estrus all year round can estrus in any season and is not limited by the lambing season. In Xinjiang, Qira black sheep, Kazakh sheep and Duolang sheep are remarkable for their unique reproductive characteristics, Kazakh sheep was a typical seasonal estrus sheep breed.^22^ Compared with traditional breeding methods, molecular marker-assisted selection has the advantages of shortening breeding cycle and improving selection efficiency. With the continuous development of research techniques and methods, studying marker loci related to reproductive traits had important guiding significance for improving sheep production efficiency.

Insulin-like peptide 3 (*INSL3*) is a constitutive product of mature, which was known to regulate cell proliferation and apoptosis in these cells through the PKA/ERK signaling pathway, highlighting its significance in sheep reproduction.^23^ Previous studies reported that *INSL3* played a key regulatory role in the differentiation and proliferation of mammalian germ cells.^24^^,^^25^ Duan et al. Reported that *INSL3* binding to RXFP2 might up-regulate the expression levels of PCNA and F-actin by activating the PLC/PKC signaling pathway to promote the proliferation and migration of germ cells, regulating reproductive performance.^26^ Additionally, Pitia discussed the role of *INSL3* in pregnant Saanen goats, demonstrating that the corpus luteum (CL) is not only the source of *INSL3* in pregnant goats, but also its target, while the reproductive organs outside the ovary are additional targets for *INSL3.*^27^ Moreover, studies in cattle had found that *INSL3* was mainly expressed in the ovary and testis, and it was found to be a major sex-specific circulating hormone in male fetuses.^28^^,^^29^ Pitia and his team had confirmed that the expression of *INSL3* and *RXFP2* in the testis of low-fertility bulls. The proportion of cells and the expression level of each cell are significantly reduced.^27^ In sheep, there are few studies on the function of *INSL3* gene. Only Anand detected the expression of *INSL3* in sheep blood by specific enzyme-linked immunosorbent assay, and found that the expression of *INSL3* in non-pregnant ewes was 4 times that of pregnant ewes.^16^ Therefore, *INSL3* and testosterone are considered to be important hormones that promote the normal decline of the testis. *INSL3* may be involved in regulating ovarian function, follicular development, ovarian hormone secretion and other reproductive processes in females. However, there were relatively few studies on the function of *INSL3* in females compared to its role in males. The seasonal reproduction of sheep mainly involves the molecular regulation of the hypothalamus-pituitary-gonad (HPG) axis and the secretion of related hormones.^30^^,^^32^ The results of this study are similar to the results of Anand et al, which can be inferred that the *INSL3* gene affects the lambing traits of sheep by affecting follicular development. In this study, we found that AA was the dominant genotype in Kazakh sheep, while AT was the dominant genotype of Qira black sheep and Duolang sheep. Kazakh sheep was a typical seasonal estrous sheep breed, while Duolang sheep and Qira black sheep were perennial estrous sheep breeds. There are significant differences. According to Table 2, the genotype distribution of *INSL3* (A100T) was in Hardy-Weinberg equilibrium (*P* > 0.05). It may be due to the fact that local herdsmen had already selected sheep, especially for high-intensity selection of reproductive traits. However, our results still indicate that the *INSL3* gene polymorphism was associated with litter size in different sheep breeds, which suggested that we could strengthen the selection intensity of this locus in future breeding.

The *WNT2B* gene is located in sheep chr.1 and belongs to the WNT protein family. It has five exons and targets the Wnt/β-catenin signaling pathway to participate in the proliferation and apoptosis of germ cells, which plays a key role in regulating female animal reproductive traits.^33^^,^^34^ Studies have found that *WNT2B* gene plays an important role in embryonic development and maintenance of cell homeostasis, but there are few studies on the expression and mechanism of *WNT2B* in sheep ovary.^35^ Ricken et al. found that *WNT2B* was expressed in follicular granulosa cells of immature rats stimulated by hormones, but *WNT2B* was expressed in ovarian surface epithelial cells, but not in granulosa cells and oocytes. It was speculated that it plays an important role in the healing of ovarian surface rupture after ovulation.^36^ Interestingly, the *WNT2B* gene was a gene that encodes a protein in the WNT signaling pathway. The *WNT2B* ligand activates the YAP**/**TAZ non-canonical WNT signaling pathway through the FZD5 receptor.^36^ The WNT signaling pathway played an important role in embryonic development, cell proliferation, differentiation, and tissue regeneration. In this study, the *WNT2B* gene had a certain effect on the seasonal reproduction and litter size of different sheep breeds, which was similar to the results of Jan. In this study, we found that the litter size with GG genotyped *WNT2B* (G126T) was significantly higher than that of GT and TT genotyped in Kazakh sheep (*P* < 0.05). The litter size with GT genotype was significantly higher than that of GG and TT genotypes in Duolang sheep and Qira black sheep (*P* < 0.05). The dominant genotype was different in sheep breeds with different estrus characteristics, which was consistent with the results of INSL3 (A100T). While, through the Hardy-Weinberg equilibrium test, it was found that the three sheep breeds deviated from the Hardy-Weinberg equilibrium (*P* < 0.05). Also, *INSL3* (A100T) and *WNT2B* (G126T) were moderately polymorphic in Kazakh sheep, Qira black sheep and Duolang sheep populations (0.25 < PIC < 0.5), which indicated that the genetic diversity of this SNP was relatively abundant in the three sheep breeds, and this locus had strong selection potential. This may be due to the fact that local herdsmen did not breed this locus, which suggests that we can improve the reproductive performance of sheep by breeding this locus in the future.

In summary, the *INSL3* (A100T) and *WNT2B* (G126T) were related to the reproductive traits of sheep. These SNPs could be used as molecular markers for perennial estrus and high fertility in sheep.

## Data Availability

We affirm that the data of the paper is true, rigorous and available. The data sets are available upon request from the corresponding author.
